# Editorial: Decoding the gut-brain axis: implications for neurodegenerative disease therapies

**DOI:** 10.3389/fnagi.2026.2015896

**Published:** 2026-10-23

**Authors:** R. M. Damian Holsinger

**Affiliations:** 1 Laboratory of Molecular Neuroscience and Dementia, School of Medical Sciences, Faculty of Medicine and Health, The University of Sydney, Camperdown, NSW, Australia; 2 Neuroscience, School of Medical Sciences, Faculty of Medicine and Health, The University of Sydney, Sydney, NSW, Australia

**Keywords:** gut dysbiosis, gut metabolites, gut microbiome (GM), gut-brain homeostasis, microbiome-based interventions, neuroinflammation, neuroprotection

Neurodegenerative diseases represent a major challenge to global healthcare, with prevalence increasing as populations age. Disorders such as Alzheimer's disease (AD), Parkinson's disease (PD), motor neuron disease, stroke-associated cognitive impairment and other age-related neurological conditions impose substantial personal, societal and economic burdens. Despite advances in understanding their molecular pathology, many therapeutic strategies remain symptomatic, with relatively few interventions capable of slowing disease progression. Consequently, there is an urgent need to identify novel biological mechanisms underlying neurodegeneration and translate these discoveries into effective preventative and therapeutic strategies.

Over the past decade, the gut-brain axis has emerged as a dynamic area of neuroscience research. This bidirectional communication network integrates the central and enteric nervous system, immune and endocrine pathways, vagal signaling, the intestinal barrier and the microorganisms inhabiting the gastrointestinal tract. The gut microbiota influences host physiology through microbial metabolites, immune regulation, intestinal integrity and neuroendocrine signaling. Disruption of these interactions has been associated with neuroinflammation, protein aggregation, mitochondrial dysfunction, oxidative stress and impaired neuronal homeostasis across multiple neurodegenerative disorders.

This systems-based perspective has stimulated interest in microbiome-targeted interventions, including dietary modification, prebiotics, probiotics, postbiotics and fecal microbiota transplantation. Simultaneously, advances in high-throughput sequencing, metabolomics, proteomics and computational biology have created new opportunities to characterize host-microbe interactions, identify biomarkers and explore therapeutic targets. The articles assembled in this Research Topic reflect both this progress and the challenges of translating gut-brain research into clinical practice. Collectively, they encompass original research, narrative and systematic reviews, a study protocol and an interventional clinical study, spanning disease mechanisms, biomarker discovery and emerging therapeutic strategies.

Several overarching themes emerge across the Research Topic. Multiple studies link gut microbial dysbiosis with motor and non-motor manifestations of neurological disease. Further, increasing attention is directed toward mechanisms involving microbial metabolites, immune activation, intestinal permeability and neural signaling. The Research Topic also demonstrates a transition from descriptive microbiome profiling toward interventions designed to modify disease-related outcomes. Finally, many contributions emphasize standardized methodologies, longitudinal investigations and multidisciplinary collaboration as essential for establishing causality and developing more precise approaches to diagnosis and treatment.

In a conceptual extension of the traditional gut-brain axis paradigm, Sun et al. introduce the gut-brain-muscle axis as a framework for exercise-mediated neuroprotection. Their review synthesizes evidence that physical activity reshapes gut microbial communities while stimulating myokines that may influence neuroinflammation, oxidative stress, synaptic plasticity and cognition. By integrating muscle biology into gut-brain communication, the authors provide a framework through which exercise-based interventions may contribute to healthy brain aging.

Broadening the concept of inter-organ communication beyond the gut, Kim et al. investigated the relationship between hepatic steatosis, skeletal muscle quality and brain aging. Using integrated CT and MRI measures in a large cohort, they found that muscle density partially mediated the association between hepatic steatosis and brain age. Although not focused directly on the gut microbiome, this work reinforces the broader concept that peripheral metabolic organs can interact through interconnected pathways influencing brain health and aging.

Recognizing diet as a major determinant of gut microbial composition, Raber and Sharpton provide a methodological framework for investigating the complex relationships among nutrition, the gut microbiome and cognition. Their review addresses dietary assessment, microbiome profiling, cognitive phenotyping and analytical approaches, including causal inference and dietary pattern derivation. These recommendations provide a roadmap for strengthening the rigor and reproducibility of diet-microbiome-cognition research.

Shifting from mechanistic insights to therapeutic intervention, Luo et al. investigated the effects of a ketogenic dietary intervention in patients with PD. The intervention was associated with changes in gut microbial composition and improvements in several clinical measures, highlighting the potential of dietary modulation as an adjunctive therapeutic approach. Although larger controlled trials are needed, these findings support further investigation of nutritional interventions as a means of modulating gut-brain interactions in PD.

Complementing dietary approaches, Hughes et al. evaluated the Lab4 probiotic as a strategy to modulate the gut microbiota in a murine model of AD. Probiotic supplementation preserved hippocampal neuronal spine density, improved recognition memory, reduced inflammatory markers and altered gut microbial composition. These preliminary findings provide preclinical support for microbiome-targeted interventions while highlighting the need for further studies to establish their mechanisms and relevance to human disease.

Broadening the therapeutic perspective, Gao et al. review gut-brain communication in stroke and evaluate the potential of microecological treatments to improve neurological recovery. Integrating evidence on prebiotics, probiotics, dietary interventions and fecal microbiota transplantation, the authors highlight how modulation of the intestinal ecosystem may influence neuroinflammation, immune regulation and post-stroke recovery. The review also emphasizes the challenges of translating microbiome-based strategies into clinical practice.

Looking beyond generalized therapeutic strategies, Clemente-Velasco et al. describe a protocol for developing a personalized dietary supplement based on the gut microbiota of patients with AD. By integrating microbiota, clinical and dietary data using artificial intelligence and network analyses, the study aims to identify individualized strategies for modulating disease-related microbial and metabolic profiles. This approach illustrates the emerging potential of precision nutrition within gut-brain axis research.

Drawing together many of the concepts explored throughout this Research Topic, Cipriano et al. review the growing evidence linking gut microbial dysbiosis with AD and dementia. By integrating findings on neuroinflammation, microbial metabolites, intestinal and blood-brain barrier dysfunction and protein aggregation, the authors illustrate the multifactorial nature of gut-brain communication in neurodegeneration. Their synthesis reinforces the value of viewing AD within a broader systems biology framework encompassing host-microbiome interactions.

Moving beyond descriptive analyses of microbial dysbiosis, Lin Z. et al. applied a multi-omics approach to investigate gut microbiota and plasma metabolites in patients with PD and mild cognitive impairment. Integrating microbial and metabolic profiles, the study identified distinct alterations associated with cognitive impairment, illustrating how metabolomics can provide insights into potential microbiota-metabolite interactions in PD. This work exemplifies the shift from taxonomic descriptions toward functional characterization of microbiome-associated metabolic changes.

Expanding the spectrum of microbiome-directed interventions, Wu et al. explored the effects of electroacupuncture in a mouse model of PD. Electroacupuncture improved motor function and intestinal barrier integrity while reshaping gut microbial composition and network structure, suggesting that its neuroprotective effects may involve modulation of gut-brain communication. These findings support the possibility that diverse interventions may converge on shared gut-brain pathways relevant to neurodegeneration.

Extending gut-brain axis research beyond cognition and motor dysfunction, Lin S. H. et al. investigated the relationship between gut microbiota composition and insomnia in PD. The observed associations suggest that alterations in the intestinal microbiota may contribute to non-motor manifestations through interconnected immune, metabolic and neurochemical pathways. These findings broaden the clinical relevance of gut-brain interactions and highlight sleep disturbance as an important target for future mechanistic and therapeutic investigations.

Building on several PD-focused contributions within this Research Topic, Tng et al. provide a comprehensive overview of shared gut microbiome signatures associated with aging and PD. By integrating evidence on microbial composition, microbial metabolites such as butyrate, intestinal barrier function and inflammatory pathways, the review highlights how age-related microbiome changes may intersect with mechanisms implicated in PD pathogenesis. It also discusses the potential of gut microbiota taxa and their metabolites as therapeutic targets, while emphasizing the need to account for aging when interpreting microbiome alterations in PD.

Liao et al. extend gut-brain axis research into the cerebrovascular space by demonstrating that obstructive sleep apnea is associated with distinct gut microbiota alterations in patients with symptomatic intracranial atherosclerotic stenosis. Their findings suggest associations between sleep-disordered breathing, microbial dysbiosis and altered microbial metabolic functions, highlighting the interplay among sleep, vascular pathology and gut microbial homeostasis.

Extending mechanistic insights into the clinical setting, Cai et al. investigated associations between gut microbial composition and imaging markers in patients with arteriosclerotic cerebral small vessel disease and idiopathic normal pressure hydrocephalus. The study identified specific microbial taxa associated with cerebral microbleeds and deep medullary vein scores, while exploratory analyses suggested their potential diagnostic value. These findings highlight the emerging potential of integrating microbiome profiling with neuroimaging to improve disease characterization and biomarker development.

Espinosa et al. systematically examine gut microbial dysbiosis across major psychiatric disorders, identifying both disorder-specific and overlapping microbial signatures. Their findings highlight the potential of microbiome profiling as a source of potential non-invasive biomarkers while emphasizing substantial heterogeneity across existing studies. The authors underscore the need for standardized methods, longitudinal validation and independent cohorts before microbiome-based diagnostic approaches can be translated into clinical practice.

Providing a fitting conclusion to the Research Topic, Yassin et al. synthesize evidence linking the gut microbiome with neurodegenerative and neuropsychiatric disorders, while highlighting key challenges to clinical translation. Their review emphasizes the need for mechanistic studies to establish causality, longitudinal human studies, standardized protocols and rigorous evaluation of efficacy, safety and durability. By highlighting the importance of validation, safety and personalized approaches, the authors underscore both the opportunities and challenges involved in translating gut microbiome research into clinical practice.

Collectively, the contributions to this Research Topic demonstrate that gut-brain axis research is moving beyond descriptive associations toward integrated models of microbial ecology, host metabolism, immune signaling and lifestyle. The next challenge is to determine which microbial signatures and biological pathways are reproducible, causally relevant and clinically actionable. Addressing this challenge will require standardized methodologies, longitudinal and multi-omics studies, well-designed interventional trials and multidisciplinary collaboration. Such efforts will be essential to determine whether microbiome-informed approaches can ultimately deliver reliable biomarkers and safe effective strategies for improving neurological health.

